# Marvels of illusion: illusion and perception in the art of Salvador Dali

**DOI:** 10.3389/fnhum.2015.00496

**Published:** 2015-09-29

**Authors:** Susana Martinez-Conde, Dave Conley, Hank Hine, Joan Kropf, Peter Tush, Andrea Ayala, Stephen L. Macknik

**Affiliations:** ^1^Ophthalmology, SUNY Downstate Medical CenterBrooklyn, NY, USA; ^2^The Dali MuseumSt. Petersburg, FL, USA

**Keywords:** surrealist movement, illusions, ambiguous images, stereopsis, anamorphic perspective, face perception, illusory contours, pareidolia

## Abstract

The surrealist movement aimed to blur the distinction between the real and the imagined. Such lack of a border between demonstrable truth and fantasy is perhaps most apparent in the art of Spanish painter Salvador Dali (1904–1989). Dali included numerous illusions in his artworks, with the intent to challenge the viewers' perceptions of reality and to enable them to see beyond the surface. The “Marvels of Illusion” exhibit, shown at The Dali Museum in St. Petersburg, FL., from June 14 to October 12, 2014, showcased Dali paintings, prints and sculptures centered on illusory themes. Here, we review the significance of illusions in Dali's art, focusing on the pieces displayed at the “Marvels of Illusion” exhibit.

## Introduction

The “Marvels of Illusion” exhibit, shown at The Dali Museum in St. Petersburg, FL., from June 14 to October 12, 2014, offered visitors a unique perceptual and cognitive experience into the world of ambiguity and illusions. The exhibition displayed a number of paintings, prints and sculptures by Salvador Dali (1904-1989), a sixteenth century piece from the School of Arcimboldo that was on loan from the Ringling Museum, and interactive demonstrations and illustrative material. Here we review the role of illusions in the art of Dali, focusing on the pieces displayed at the “Marvels of Illusion” exhibit.

Illusions are noted as the disconnect between physical reality and subjective perception (Martinez-Conde and Macknik, 2010). When experiencing a visual illusion, we may see something that is not there in reality, fail to see something that is, or more generally see something different from what is there. Due to this disconnect between perception and reality, visual illusions exemplify how the brain fails to re-create the physical world, and provide vision scientists with substantial tools to apply to the study of the neural underpinnings of perception.

Throughout history, artists and researchers have utilized illusions with the aim of understanding perception. Many years before scientists began studying neuronal properties, artists devised multiple techniques to trick the brain into believing that a flat canvas had depth or that a sequences of brushstrokes was in fact a still life. Factors such as brightness, color, shading, and eye movements, among other contributors, can powerfully affect what we see.

Salvador Dali intuited that what we construe visually as reality is the product of the habits of the mind, more than of the eye. He understood that we create an ordered or disordered world from intermittent and incomplete retinal information processed by our mind's experiences, desires and apprehensions. Thus, Dali's artworks challenge the viewers' perceptions of reality and enable them to see beyond the surface. Visual illusions, present in many of the painter's artworks, include numerous examples of perceptual completion and ambiguous images.

## Illusory contours and filling-in illusions in Dali's art

Our brain makes up a large fraction of what we perceive. High-resolution vision is limited to the center of our eyes—about a tenth of a percent of the entirety of our visual field—, but we perceive the whole visual field as a high-resolution, focused, perfectly formed image. This is a grand illusion that results from the joint action of the neural systems responsible for our vision and eye movements.

Various perceptual rules, such as the Gestalt laws conceptualized in the late 19th and early 20th centuries, govern the way our brains fill in incomplete information. For instance, the Gestalt Principle of Closure says that our perception will group individual elements as a whole (rather than consider them as separate from each other) if they seem to complete an entity. The Kanizsa triangle illusion appears as a ghostly triangle partially superimposed on three circles at the triangle's vertices. We perceive the triangle, rather just than the three Pac-men that are actually present, because our brain overlays the shape of a triangle on an extremely limited field of data. The illusory triangle manages to look slightly whiter than the background, though it is in reality the same shade. A great deal of our everyday experience consists of similar feats of filling in perceptual and cognitive gaps, where we use what we know about the world to imagine what we do not know.

Our visual system is ingrained with the ability to detect and process faces rapidly and with efficiency, even with few details. Even infants look at basic depictions of faces for longer times than they explore similar cartoonish faces in which the eyes and other features are scrambled. The neurons responsible for our refined “face sense” lie in the fusiform gyrus or fusiform face area, a brain region that becomes active not only when we detect an actual face, but also when we perceive an illusory or imaginary face. Meng et al. recently found that, whereas both faces and objects that look like faces activate the left fusiform gyrus, real faces activate the right fusiform gyrus much more strongly than look-alikes (Meng et al., 2012).

Trauma or lesions to the fusiform face area result in a prosopagnosia, or face blindness. But even people with standard face-recognition skills are susceptible to various face perception illusions. Many of these occur when the visual system fills in the gaps to create a complete face from scarce visual content.

“Face pareidolia” refers to our visual system's predisposition to find faces in accidental or vague visual information. Common examples are finding faces on the fronts of cars and buildings. This phenomenon results from face-recognition circuits that are constantly at work to find a face in the crowd. Our brain's aptitude to find meaning, united with an outstanding skill for face detection, can lead to spectacular cases of pareidolia. A grilled-cheese sandwich, with an image resembling Virgin Mary burned into the bread, sold on eBay for $28,000 (Martinez-Conde and Macknik, 2012).

The brain's ability to fabricate links among things that are in reality unconnected is essential to the “paranoiac-critical method” artistic method invented by Dali. (In fact, paranoia and pareidolia share a common etymology, from the Greek *para*- for “instead of” and -*oid*, -*oeides*, or -*eidos* for “form”).

### Paranoia—oil on canvas, 1935–36

**Figure 1:**
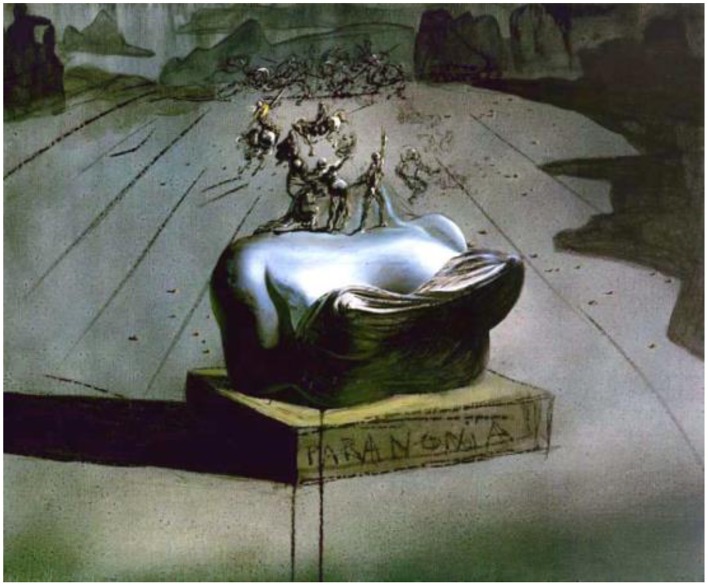
**Paranoia, by Salvador Dali, Oil on canvas, 1935–1936**. ^©^Salvador Dali. Fundación Gala-Salvador Dali (Artist Rights Society), 2015. Collection of the Salvador Dali Museum, Inc., St. Petersburg, FL, 2015. Reproduced with kind permission from the Salvador Dali Museum, Inc.

*Paranoia* provides a striking example of an illusory contour resulting from filling-in processes. A battle scene reminiscent of some of Leonardo Da Vinci's sketches hovers over a bust set on a pedestal. The bust is headless, yet we perceive a head (Figure 1).

The small figures that appear to be standing on or behind the woman's neck form her chin, mouth, and nose. In the distance, groups of men on horseback form the eyes and hairline. The brain then fills in the missing lines and contours of the woman's face (Cox et al., 2004). Facial recognition is a dominant perceptual function, so the brain easily completes the head despite having to fabricate most of the information.

The woman's face can be seen more easily by squinting our eyes to blur the distinct edges of the small figures. Interestingly, there is also a double image in the face. Some people can see a sweet woman with downcast eyes, while others see a wild-eyed woman with a sinister smile (see “Ambiguous Illusions” section for more examples of perceptual ambiguity in Dali's art).

Paranoia pays homage to Leonardo, not only in the depiction of the battle scene, but also in following his advice to find perceptual patterns in meaningless objects: “*…stop sometimes and look into the stains of walls, or ashes, or a fire, or clouds, or mud, or like places, in which, if you consider them well, you may find really marvelous ideas,”* Leonardo wrote in his notebooks.

### The Madonna of the birds—watercolor on paper, 1943

**Figure 2:**
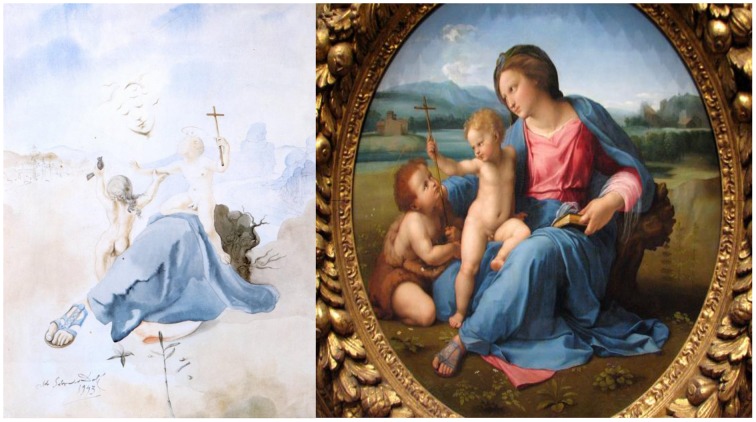
**Left:** The Madonna of the Birds, by Salvador Dali, Watercolor on paper, 1943. ^©^Salvador Dali. Fundación Gala-Salvador Dali (Artist Rights Society), 2015. Collection of the Salvador Dali Museum, Inc., St. Petersburg, FL, 2015. Reproduced with kind permission from the Salvador Dali Museum, Inc. **Right:** Alba Madonna, by Raphael, c. 1511.

The *Madonna of the Birds* watercolor (Figure 2, left) is based on the Italian High Renaissance artist Raphael's (1483-1520) *Alba Madonna*, c. 1511 (Figure 2, right). In Dali's version, the torso is only suggested, and the face is formed by a flock of birds. Neurons in our visual cortex connect the shapes of the individual birds, to form the illusory contour of the expected but missing head, as well as the hair, eyes, mouth and chin. Dali kept the hue and value of the birds subdued, to merely hint at the face.

Connecting the head to the body requires a larger perceptual effort than filling in the face. The torso gap is large, and the lack of details and suggestive lines in the bodice challenges our visual system to generate the perception of a whole upper body where we know it should be.

Dali's borrows the compositional arrangement of Raphael's original. In Dali's version, the Christ child, identified by a halo, holds the slender cross while seated on the Virgin's lap. Another child, John the Baptist, reaches up to face the Madonna with a small bird in hand. Dali replicates the sandal worn by Raphael's Madonna.

### La soif (thirst)—ink and gouache on paper, 1965

**Figure 3:**
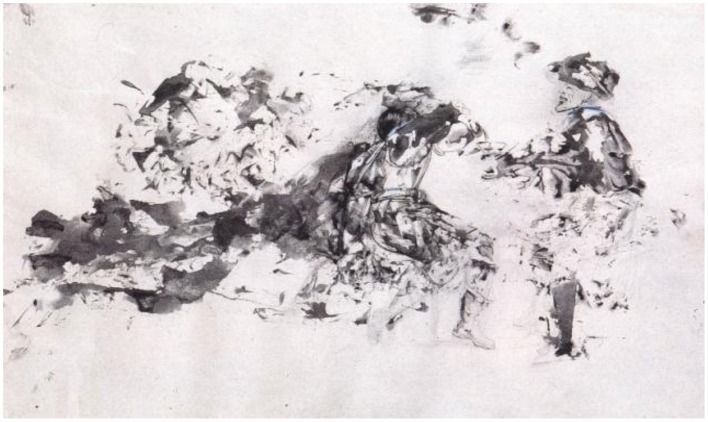
**La Soif, by Salvador Dali, Ink and gouache on paper, 1965**. ^©^Salvador Dali. Fundación Gala-Salvador Dali (Artist Rights Society), 2015. Collection of the Salvador Dali Museum, Inc., St. Petersburg, FL, 2015. Reproduced with kind permission from the Salvador Dali Museum, Inc.

In *Thirst*, Dali either used decalcomania (folding a piece of paper with wet gouache inside, and then peeling it open) or took an ink-soaked cloth and pressed it onto the surface of the paper. Within the ink and gouache blotches he visualized two Renaissance figures in period clothing, one serving wine to the other (Figure 3). He then drew line and shape fragments and left it to our imagination to complete the implied presence of objects in the scene. The trousers of the person in the right are little more than blotches of ink, and yet, in context, our perceptual processes fill in the missing information so we recognize the overall shape as a piece of clothing.

Dali created his own system of observation, his celebrated paranoic-critical method, in which the artist could look at any object and see another. In *The Conquest of the Irrational*, Dali described that his aim was to “*materialize the images of concrete irrationality with the most imperialist fury of precision”* (Dali, 1935). Dali's goal was to achieve images that could not be analyzed or diminished by rational logic.

Dali was very familiar with Leonardo da Vinci's notes in his *Treatise on Painting*, which contained the following advice on seeing hidden images: “*look at certain walls dirtied with various stains…you will be able to see various battles and figures…and strange expressions on faces, and costumes, and an infinite number of things”* (Da Vinci, 1956). Dali's ability to identify different images within a given configuration allowed him to perceive reality from a fresh perspective.

## Ambiguous illusions in Dali's art

Dali's art includes frequent examples of ambiguous illusions, where the brain interprets the same picture in two mutually exclusive ways. The physical object is unchanged, yet it produces two (or more) contradictory percepts. By creating accessible double images, Dali asks us to reconsider on a fundamental scale our constructs of reality.

### Femme-Cheval—ink, 1933

**Figure 4:**
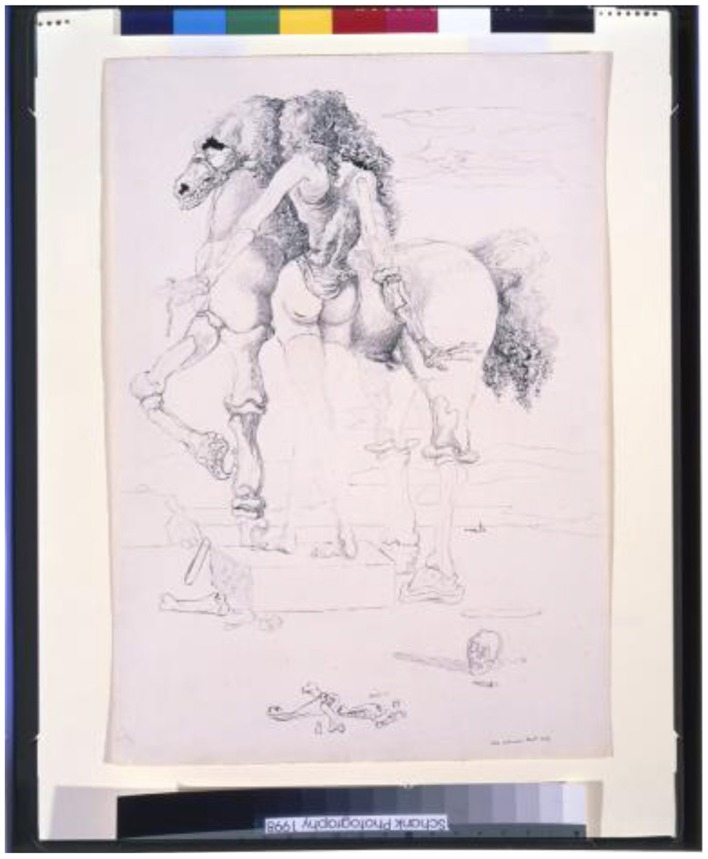
**Femme-Cheval, by Salvador Dali, Ink, 1933**. ^©^Salvador Dali. Fundación Gala-Salvador Dali (Artist Rights Society), 2015. Collection of the Salvador Dali Museum, Inc., St. Petersburg, FL, 2015. Reproduced with kind permission from the Salvador Dali Museum, Inc.

Dali's *Femme-Cheval* challenges the viewer to determine if the two drawn figures are part of one image or another, and to guess where one figure ends and the other begins. The intermingling of the mane and the woman's hair, or the woman's legs that are rendered so faintly that they disappear, causes perceptual ambiguity (Figure 4). Our brain also fills in incomplete or missing information for each of the perceptual interpretations. Many of the illusions that we discuss here as ambiguous also include significant illusory contours and filling-in/perceptual completion, and vice versa. The various illusory components play off, and enhance, each other.

According to Dali's 1930 essay L'Ane pourri, “*The double image (an example of which might be the image of a horse that is at the same time is the image of a woman) may be extended, continuing the paranoiac process, with the existence of another obsessive idea being sufficient for the emergence of a third image […] and thus in succession until [the number of images is] limited only by the extent of the mind's degree of paranoiac capacity (Dali*, *1998a**).”*

### Nieuw amsterdam—bronze sculpture painted with oil and added metal, 1974

**Figure 5:**
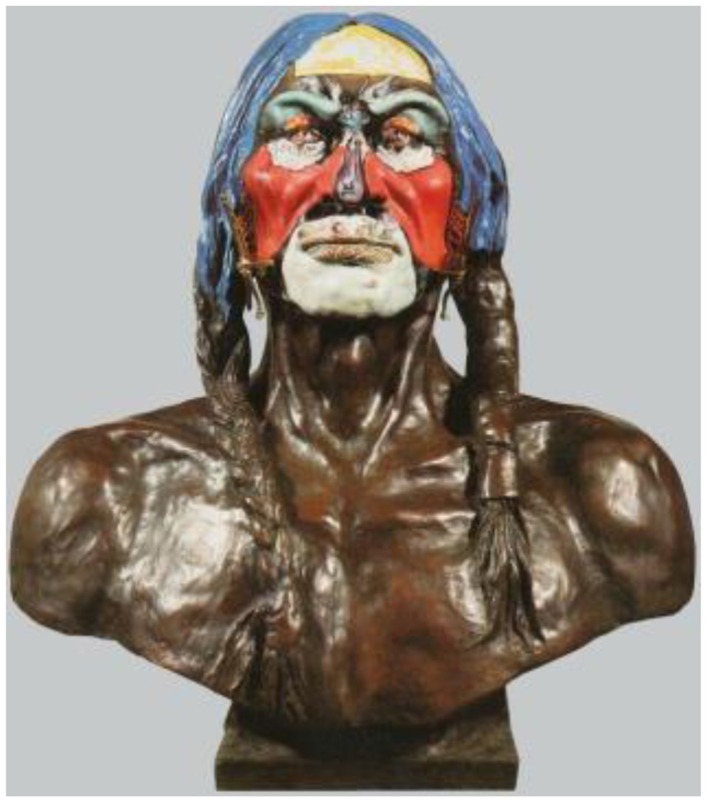
**Nieuw Amsterdam, by Salvador Dali, Bronze sculpture painted with oil & added metal, 1974**. ^©^Salvador Dali. Fundación Gala-Salvador Dali (Artist Rights Society), 2015. Collection of the Salvador Dali Museum, Inc., St. Petersburg, FL, 2015. Reproduced with kind permission from the Salvador Dali Museum, Inc.

Dali painted directly onto a copy of the famous nineteenth-century bronze bust of *White Eagle* (1899) by the American sculptor, Charles Schreyvogel (1861–1912). In doing so, Dali transforms the bust into a three-dimensional scene as envisioned by his paranoic-critical method (Figure 5). Although technically this may be classified as an ambiguous illusion, the ambiguity between competing perceptions (scene vs. face) is more subtle than in other artworks. The ambiguity arises when Dali uses the facial features of the sculpture to define a scene: the outline of White Eagle's eyes form the faces, the cheekbone shadowing forms the arms, the chin forms the table.

The painted scene features two Dutch merchants at a table. On White Eagle's forehead is a wall map surrounded by blue drapery. The red capes of the merchants cover the cheeks while their plumed hats define the eyebrows. The merchants are seated on a divided miniature metal chair, which is attached to the bust. The figures are toasting a Coca-Cola bottle, the presence of which combines a modern symbol with the otherwise traditional embellishment of the sculpture. The chief's chin is transformed into a table top with the lips becoming a basket of fruit.

### Old age, adolescence, infancy (the three ages)—oil on canvas, 1940

**Figure 6:**
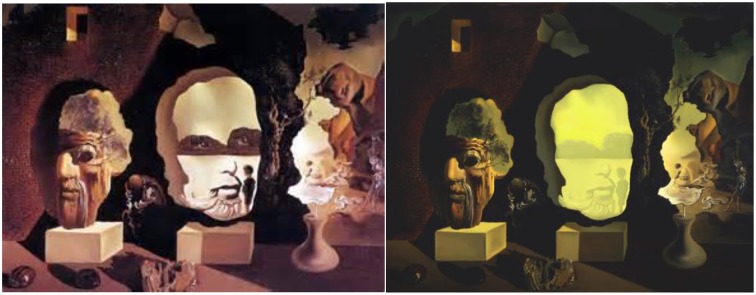
**Left:** Old Age, Adolescence, Infancy (The Three Ages), by Salvador Dali, Oil on canvas, 1940. **Right:** The Three Ages, with Adolescence subdued. ^©^Salvador Dali. Fundación Gala-Salvador Dali (Artist Rights Society), 2015. Collection of the Salvador Dali Museum, Inc., St. Petersburg, FL, 2015. Reproduced with kind permission from the Salvador Dali Museum, Inc.

In The Three Ages, cues of textures and apparent openings suggest a plausible wall of arches through which we see distant scenes. Competing with that interpretation, our visual system's bias for face detection, and the high-contrast edges that define the shapes of the heads, indicate to our brain that we are seeing faces against a dark background (Figure 6, left).

Dali's selection of lighter hues and shading values for the three “faces” leads our perception to make sense of the scene by grouping these areas as facial entities, separate from the dark of the surrounding “background.” But Dali may not have achieved as much perceptual ambiguity as he sought. For Old Age and Adolescence, the faces dominate the ambiguity struggle, partially due to Dali's choice of almost saturated (solid) dark hues with high contrast edges for the details of the faces, which sets up a strong preference in our brain for the facial interpretation. It is easier to see the Old Age and Adolescence faces (left and middle) than the arches and the scenes in the distance. Conversely, Infancy (right) blends more subtly with the opposing image of fisherwomen mending nets, resulting in greater ambiguity.

Dramatically subduing the figures and hills in Adolescence sets up a stronger ambiguity between figure and background where our mind now can perceive an opening in the wall with greater ease than in the non-subdued image (Figure 6, right).

### Study for “the three ages”—pencil on paper, 1940

**Figure 7:**
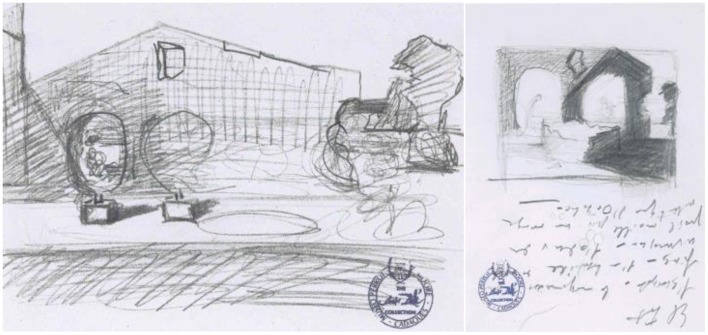
**Study for “The Three Ages,” by Salvador Dali, Pencil on paper, 1940**. ^©^Salvador Dali. Fundación Gala-Salvador Dali (Artist Rights Society), 2015. Collection of the Salvador Dali Museum, Inc., St. Petersburg, FL, 2015. Reproduced with kind permission from the Salvador Dali Museum, Inc.

As Dali prepared for *The Three Ages*, he sketched figures and experimented with shading, size, and other elements that would be in the final image (Figure 7). Seeing through the eyes of his paranoic-critical view of the world he searched for the elements that would best induce perceptual ambiguity.

### Study for “disappearing image”—charcoal on paper, 1939

**Figure 8:**
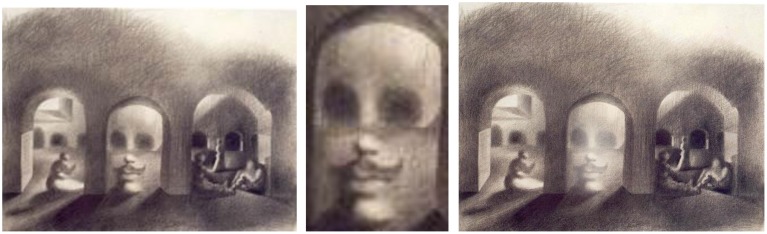
**Left:** Study for “Disappearing Image,” by Salvador Dali, Charcoal on paper, 1939. **Center:** Study for Disappearing Images: enlarged face. **Right:** Study modified. ^©^Salvador Dali. Fundación Gala-Salvador Dali (Artist Rights Society), 2015. Collection of the Salvador Dali Museum, Inc., St. Petersburg, FL, 2015. Reproduced with kind permission from the Salvador Dali Museum, Inc.

Dali's preliminary study for *The Three Ages* explores the development of an ambiguous illusion. On the one hand, we can easily see through the archways, past the figures to the courtyard beyond. But we can also identify objects that look like eyes, mouths, and heads that are strong triggers for our face detection neurons (Figure 8, left). The confusion stretches our mind's ability to make sense of what we are really seeing.

While this study has an overall similarity to the final oil painting, the specific faces are different. In the oil painting, there is an allegorical progression of the three stages of man: infancy, adolescence, and old age (from right to left). In this earlier study, the specific faces are much less precise, and the order of the ages is reversed. An indeterminate face appears on the left, possibly a child close to adolescence, and an older looking adolescent bearing a mustache is in the center. The face on the right is a skull, perhaps representing death, which Dali abandoned in the finished canvas.

Squinting or stepping away from the image blurs the fine details defining the objects and people inside the arches, allowing the face interpretation to dominate our perception instead.

Dali experimented very carefully with sketching shapes, shading, edge details, and placement to set up the double images. We can see how these elements matter by zooming in on the center image to eliminate the presence of a wall. The result is more obviously a face (Figure 8, center).

Conversely, softening the intensity of the features in the middle face degrades the cues for face detection and allows us to more easily perceive the arch as a structure (Figure 8, right).

### Changes in great masterpieces, rembrandt—lithograph, 1974

**Figure 9:**
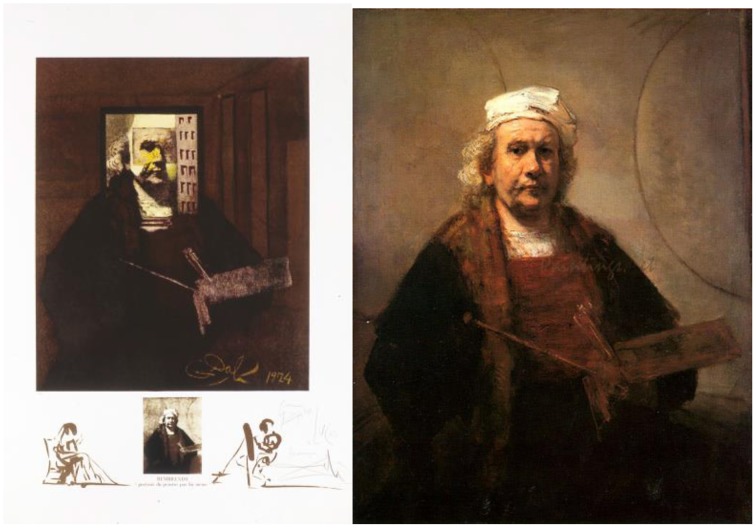
**Left:** Changes in Great Masterpieces, Rembrandt, by Salvador Dali, Lithograph, 1974. ^©^Salvador Dali. Fundación Gala-Salvador Dali (Artist Rights Society), 2015. Collection of the Salvador Dali Museum, Inc., St. Petersburg, FL, 2015. Reproduced with kind permission from the Salvador Dali Museum, Inc. **Right:** Rembrandt's original: Dali saw a receding dark hallway to a woman in a lit room through an open door.

Dali paid tribute to the old masters, such as Raphael, Rembrandt, Ingres, Vermeer, and Velazquez, through his paranoiac-critical view of the world. In *Changes in Great Masterpieces, Rembrandt* (1974), Dali saw an open door and receding dark hallway in the self-portrait of Rembrandt van Rijn (1606–1669), and set up a double image by using contrast and shape cues (Figure 9).

To create perceptual ambiguity, Dali reduced the overall value of Rembrandt's image to create a much darker version of the portrait. This allowed him to transform the otherwise flat background into a receding wall meeting the floor at what would be Rembrandt's arm. These cues of perspective produce the perception that the hallway recedes into the distance. The brighter opening suggests a lit room beyond the wall. An open door and the hard edges of a doorframe complete that interpretation.

The ambiguity lies in how our perception switches back and forth between Rembrandt's face and the hallway scene. As is often the case in this type of illusion, focusing on the close-up details helps the perception of one image (woman in room at end of hall), while stepping back to view the whole reveals the larger portrait. Squinting one's eyes also helps to perceive the portrait as the dominant scene, by blurring the edges and boundaries of the fine details of the door and hallway.

### Transformation of *Antiques* magazine cover into the apparition of face—gouache on magazine cover, 1974

Dali had a vision of a face on the original cover of Antiques Magazine. He had been fascinated with camouflage and mimicry in nature since he was a child (see also “*Tres Picos*” for Dali's particular interpretation of camouflage in the natural world). This fascination influenced the invisible and paranoic images that inhabit his paintings.

**Figure 10:**
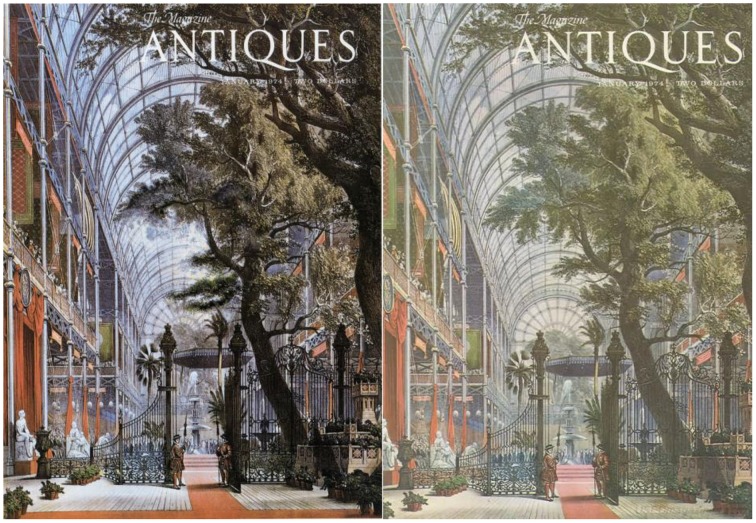
**Left:** Transformation of *Antiques* Magazine Cover into the Apparition of Face, by Salvador Dali, Gouache on magazine cover, 1974. ^©^Salvador Dali. Fundación Gala-Salvador Dali (Artist Rights Society), 2015. Collection of the Salvador Dali Museum, Inc., St. Petersburg, FL, 2015. Reproduced with kind permission from the Salvador Dali Museum, Inc. **Right:** Antiques original 1974 cover for comparison.

In the *Transformation of Antiques Magazine Cover*…, Dali creates an ambiguous illusion where our visual system struggles between the alternate and incompatible perceptions of a face and a scene within the Crystal Palace mall. Looking at the image closely, we may focus on the easily recognizable branches and leaves of the tree. Or we can look at the lines and shading of the glass ceiling, and identify a plausible arched structure fading into the distance (Figure 10).

But when we focus on the image as a whole, especially when we step back, the strongly contrasting edges of the dark tree against the light background provide us with sufficient cues to discover a face. The shading and changes of tone within the backdrop also shape our perception of the curvature of the face and the protruding eyebrows, nose and lips. Even with the obvious face, however, the scene of a tree in a mall is not lost, and our mind switches back and forth between face and scene interpretations.

### Illustration for “tres picos”—watercolor and ink conversion of print, 1955

**Figure 11:**
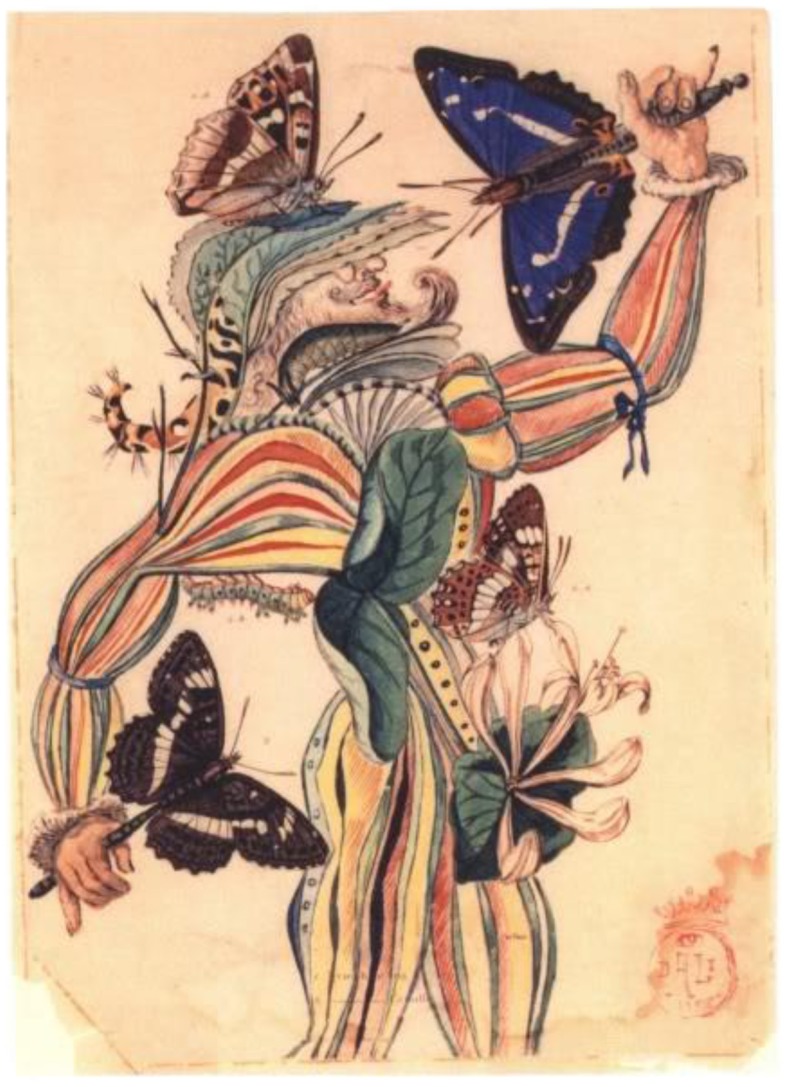
**Illustration for “Tres Picos,” by Salvador Dali, Watercolor and ink conversion of print, 1955**. ^©^Salvador Dali. Fundación Gala-Salvador Dali (Artist Rights Society), 2015. Collection of the Salvador Dali Museum, Inc., St. Petersburg, FL, 2015. Reproduced with kind permission from the Salvador Dali Museum, Inc.

Dali wrote in 1942 the *Total Camouflage of Total War*, in which he states, “*The discovery of ‘invisible images’ was certainly my destiny (Dali*, *1998b**)*.” His skill of employing a variety of techniques to create unusual effects in his art is based on his ability of “seeing things differently.” Dali's capacity to “read” other configurations in illustrations by other artists prompted this interpretation of *Tres Picos* (Figure 11).

Dali's use of the butterfly highlights his appreciation for the insect's natural beauty and his attraction to it as a symbol of metamorphosis. The ambiguity in this illusion comes from the costumed man being both a man, and a configuration of butterflies, larva, and plants. The male and the female of the Apatura Iris (Purple Emperor) species of butterfly can be perceived as either butterflies, or as fans or masks for a formal masquerade. A caterpillar curling into a leaf to pupate forms the man's tricorner hat, while a butterfly alighting on top could also be a hat plume.

Dali and the surrealist movement rediscovered the amusing and reality-stretching artwork of the Italian painter Giuseppe Arcimboldo (1527–1593), which likely inspired pieces such as *Tres Picos*. Arcimboldo's portraits are ambiguous illusions because our perceptions dance between seeing a face and a collection of fruits and vegetables. Both are familiar objects to our brain and Arcimboldo controlled the variables of the painting to keep it intriguingly ambiguous.

**Figure 12:**
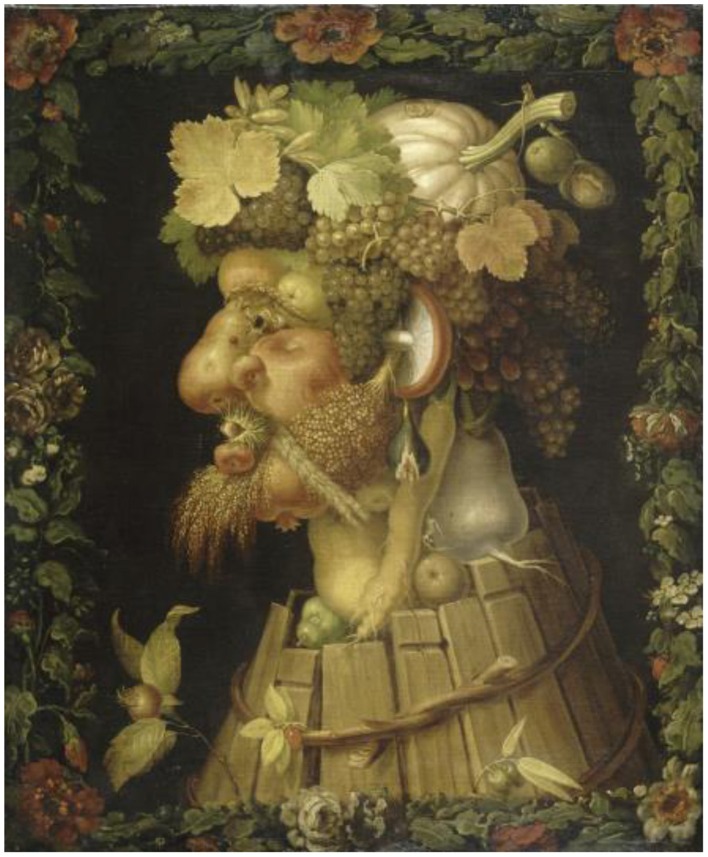
**Autumn, by Arcimboldo**.

The pear that defines the nose in Arcimboldo's *Autumn* (Figure 12) is not a bright yellow or greenish as pears can be. Instead, the hue (color) is chosen to be yellow-orange, with muted tones. Gradations in value, especially increasing at edges, suggest contour, mass, and dimension. It is a pear to our perception: but it is also a plausible nose. Each fruit or vegetable is thus chosen to define the color and contour of its part of the portrait. They blend in such a mimicking fashion that our mind has to “look twice” to make sense of what it is seeing.

The brain manufactures object representations from discrete features, like line fragments and minute color patches. We perceive a nose in *Autumn*, not due to a retinal neuron that processes noses, but to a myriad photoreceptors that react to the various shades of luminance and color in that region of the painting. Cortical circuits subsequently match that information to our neural template for noses. The same photoreceptor output also allows other cortical neurons to discern the pears, grapes, and leaves, making images like these so delightful to contemplate.

As is often the case with this type of ambiguous illusion (see *Gala Contemplating the Mediterranean*…for a spectacular example), stepping back and squinting our eyes homogenizes the values, de-saturates the hues, and blurs the edges that our brain uses to define details in shapes, allowing us to see the face as a whole, rather than as a collection of fruits and vegetables.

Whereas many of Arcimboldo's portraits are examples of mosaicism, where a large object such as a hat is made up of smaller ones such as grapes and leaves, Dali's ambiguous images usually involve reversals of figure and background.

### The sheep—gouache on a chromolithography by schenck, 1942

**Figure 13:**
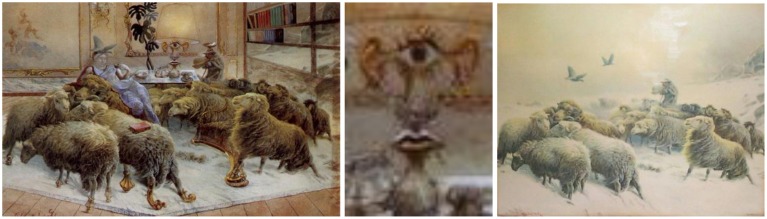
**Left:** The Sheep, by Salvador Dali, Gouache on a chromolithography by Schenck, 1942. **Center:** Lamp detail. ^©^Salvador Dali. Fundación Gala-Salvador Dali (Artist Rights Society), 2015. Collection of the Salvador Dali Museum, Inc., St. Petersburg, FL, 2015. Reproduced with kind permission from the Salvador Dali Museum, Inc. **Right:** *Lost on the Mountain*, by Albrecht Schenck, c. 1873/84.

*The Sheep* demonstrates Dali's capacity to scrutinize and reconfigure the visual world, and then present this new vision for others to see. Dali applies gouache to a reproduction of Albrecht Schenck's chromolithograph, *Lost on the Mountain* (c. 1873/84) to add or blot out details, blurring the line between the original and his additions. Compare Schenck's original *Lost on the Mountain* (Figure 13, right) to Dali's *The Sheep* (Figure 13, left).

As we look at *The Sheep*, the scene surprises our mind with a number of ambiguous images. We recognize a familiar herd of sheep but they appear to be inside a room, and be part of the furniture. Thus we perceive something that fluctuates between furnishings and a group of animals.

The face of the woman also features an ambiguous illusion. The face is subtle, which could almost be texturing on the wall. Although the two interpretations alternate in our perception, the context of the woman's body in repose and the numerous facial details bias our facial recognition system toward perceiving a face.

Close examination of the lamp on the table (Figure 13, center) reveals an eye, ears, nose, mouth and neck, which together with the lamp's shade, provide our visual circuits with plenty of cues to fill in the information that is missing and thus match our neural template for a face.

### La lecon d'anatomie (the anatomy lesson)—ink on paper, 1965

**Figure 14:**
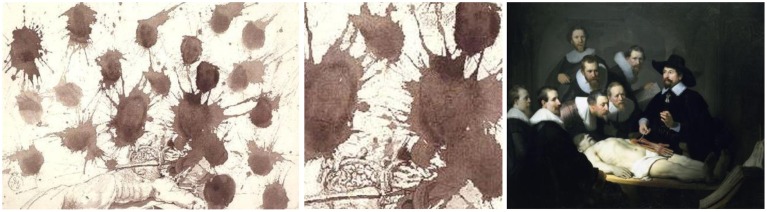
**Left:** La Lecon d'Anatomie (The Anatomy Lesson), by Salvador Dali, Ink on paper, 1965. **Center:** Inkblots detail. ^©^Salvador Dali. Fundación Gala-Salvador Dali (Artist Rights Society), 2015. Collection of the Salvador Dali Museum, Inc., St. Petersburg, FL, 2015. Reproduced with kind permission from the Salvador Dali Museum, Inc. **Right:** *The Anatomy Lesson of Dr. Jan Deyman*, by Rembrandt, 1656.

Dali reinterpreted Rembrandt's *The Anatomy Lesson of Dr. Jan Deyman, 1656* (Figure 14). The original painting was based on the public dissection of an executed criminal at the Anatomy Theater of the Guild of Surgeons in Amsterdam. Wealthy citizens and physicians observed the procedure (Figure 14, right).

Dali's ink composition utilizes elements similar to the original with seven inkblots bearing subtle figures of Diego Velazquez (1599–1660), Christ, and Dali himself (Figure 14, left). The cadaver is angled with Velazquez to the right, using a saw to open the cranium exposing the brain of the subject. The face of Christ is to the left with his eyes closed.

In keeping the inked images of faces subtle, Dali carefully crafted an ambiguous illusion where our mind juggles back and forth between seeing faces within the blots (Figure 14, center), or seeing the dark inkblots themselves contrasting sharply against the white paper as a set, perhaps suggesting blood spatter from the body laying at the bottom of the scene.

### Decalcomania—watercolor on black paper, 1936

The neural bases of imagination are poorly understood. Dali's imagination, perhaps more fertile than most, was driven by his paranoic-critical methodology of seeing things in surprising ways. Dali, and other surrealist artists of the time, experimented with Oscar Dominguez's (1906–1957) decalcomania technique of folding a piece of paper with wet gouache and peeling it back slowly to reveal a pattern for the artist to discover a spontaneous reality within.

**Figure 15:**
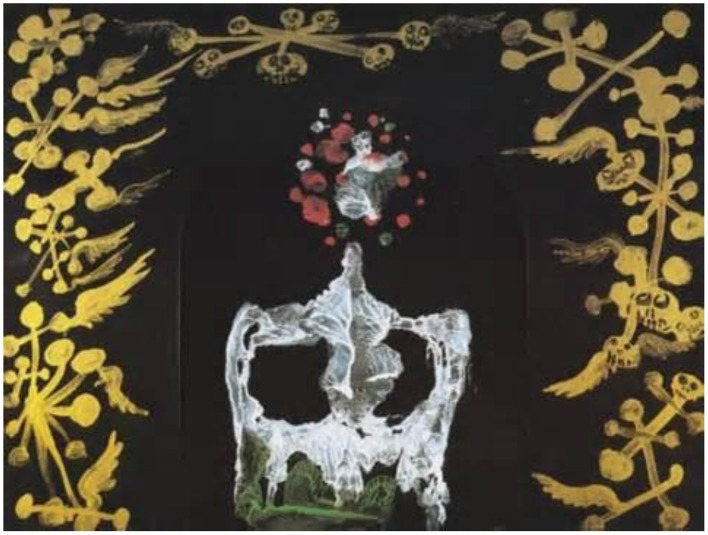
**Decalcomania, by Salvador Dali, Watercolor on black paper, 1936**. ^©^Salvador Dali. Fundación Gala-Salvador Dali (Artist Rights Society), 2015. Collection of the Salvador Dali Museum, Inc., St. Petersburg, FL, 2015. Reproduced with kind permission from the Salvador Dali Museum, Inc.

Our brain is wired to find meaning and structure around us, so we struggle to make sense of images like Dali's *Decalcomania* (Figure 15). Edge and contour detection starts with our retinal neurons, which then pass on that information to later stages of visual processing in the brain, until it reaches the cortical areas responsible for our perception of shape and color. Along the way, we compare the incoming visual information to known objects in our memories. If it makes sense, like perhaps the haunting skeletal shape of a female with red hair around a face, we accept it. If it does not, we may conjure up alternative interpretations.

The high contrast forms bring Rorschach inkblot tests to mind, and similarly prompt our imagination to identify specific shapes. The symmetry helps the perceptual association to similar objects, as many things in the natural world are symmetrical. Although this is a type of ambiguous illusion, here Dali has not embedded two competing images that confuse the brain. The ambiguity lies in the lack of genuine images, so the brain is challenged to conjure any number of rivalrous hypotheses. This is also a filling-in illusion: our visual neurons fill in and complete the positive and negative spaces to help us resolve familiar objects.

### Head of donkey—ink, 1936

**Figure 16:**
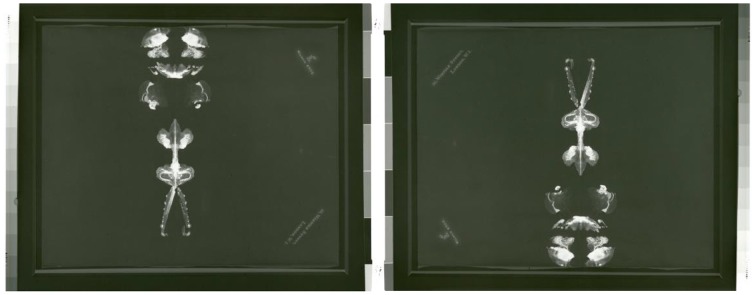
**Left:** Head of Donkey, by Salvador Dali, Ink, 1936. **Right:** Rotated to see the insect. ^©^Salvador Dali, Fundación Gala-Salvador Dali (Artist Rights Society), 2015. Collection of the Salvador Dali Museum, Inc., St. Petersburg, FL, 2015. Reproduced with kind permission from the Salvador Dali Museum, Inc.

Dali explored the decalcomania process of gouache on folded paper (in this case, stationary from the house of Edward James, Dali's patron), to then open it and let his paranoic-critical imagination look for images within. Rorschach ink-blot in nature, images like these provoke our imagination to look for familiar shapes or meaningful images within them. In this particular case, the image looked insect-like when viewed one way, but became the *Head of a Donkey* when turned upside-down (Figure 16).

**Figure 17:**
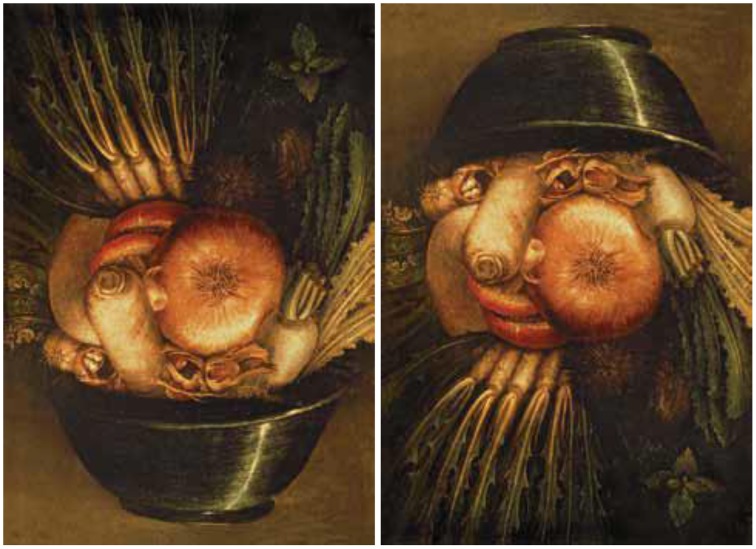
**This still life by Arcimboldo depicts a bowl of vegetables (left) that becomes a fanciful portrayal of a man's head, capped with a bowler hat, when turned upside down (right)**.

Our brain is wired to notice, identify and discriminate facial expressions and features from minimum data. This capacity is essential to our social interactions and the reason we attribute emotions and personality to objects such as rudimentary masks and the front ends of vehicles. In that case, why don't we perceive the donkey's face when we rotate the image vertically? The reason is that the neural processes that allow us to see faces quickly and effortlessly are optimized to detect right-side-up faces, so upside-down faces are harder to distinguish (Figure 17).

### Gala contemplating the mediterranean sea which at twenty meters becomes the face of abraham lincoln—homage to rothko—2nd version, oil on canvas, 1976

**Figure 18:**
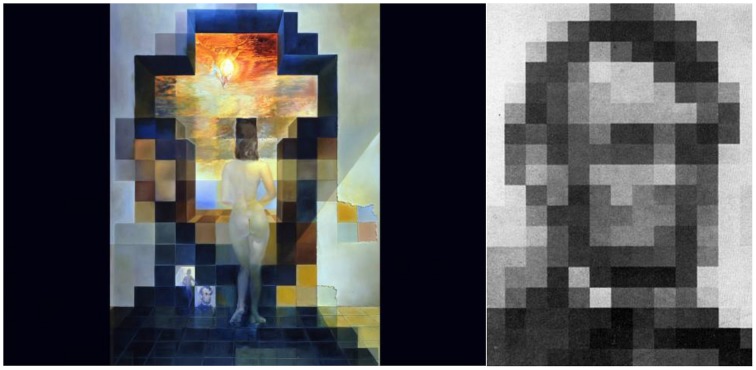
**Left:** Gala Contemplating the Mediterranean Sea Which at 20 M Becomes the Face of Abraham Lincoln—Homage to Rothko, by Salvador Dali. 2nd version, oil on canvas, 1976. ^©^Salvador Dali. Fundación Gala-Salvador Dali (Artist Rights Society), 2015. Collection of the Salvador Dali Museum, Inc., St. Petersburg, FL, 2015. Reproduced with kind permission from the Salvador Dali Museum, Inc. **Right:** Harmon's original 16 × 16 gray-scale block averaging image of Lincoln. Dali pays tribute to Harmon by including this image as one of the cells in the lower left of his painting.

Another way to create ambiguous illusions is by pitting high-resolution fine detail against low-resolution overriding shapes, as in Dali's *Gala Contemplating the Mediterranean Sea, which at Twenty Meters Becomes the Portrait of Abraham Lincoln*, one of the painter's finest ambiguous illusions (Figure 18).

Dali created this piece after reading about Leon D. Harmon's groundbreaking work, published in 1973 in *Scientific American* with the title “*The Recognition of Faces.”* Harmon had produced “block averaging” renderings of a picture of Abraham Lincoln, taken from a $5 bill. Block averaging entails breaking down an image into blocks of a grid, and filling each block with its average gray-scale value; in other words, assigning a single tone to each pixel. Harmon found that 16 × 16 (256 total) was the smallest number of blocks necessary to recognize a face (Harmon, 1973).

The homage to Mark Rothko (1903–1970) paid tribute to the abstract expressionist, who had recently committed suicide. Dali used blocks of color in hues that bring to mind Rothko's “color field” paintings.

Gala's figure is comprised of high spatial frequencies, whereas Lincoln's face contains low spatial frequencies. When we stand close we focus on the keen differences of value and hue, and the other high-spatial frequency particulars, so we notice a crucifixion rendered in heavy impasto in the sky, and Gala staring out a cruciform window facing the sea. Such high spatial frequencies, which govern our perception at close range, obscure Lincoln's face.

As we stand farther away (20 m) from the painting, the low spatial frequencies dominate our perception instead: we now see the coarser, less intricate elements of the scene, rather subtle details such as Gala's outline and the edges of the large blocks. We no longer witness Gala; the high spatial frequencies that define her body blend into the surrounding region (which has similar light values to those in Gala's figure), leaving us just with the general low spatial frequency shadings and shapes that constitute Lincoln's face. Squinting our eyes near the painting also helps us smear and soften the edges, by removing the high spatial frequency information and revealing the face “hidden” in the low frequencies. Dali's selection of hues, values, tones, textures, and saturation for the sea, clouds, and Gala's body thus become appropriate shading to perceive Lincoln's skin.

Once we start to recognize Lincoln's visage, our face-processing neurons contribute additional details to fill in the image. After we connect Lincoln's face to a specific group of squares, it is hard to cease seeing it. Dali and Harmon did not pick Lincoln at random: we identify familiar faces more easily than unfamiliar ones.

Re-approaching the painting makes Lincoln disappear and Gala reappear, as the painting becomes once again subjugated to fine details.

Many of Dali's artworks involving double images rely on the interplay of high and low spatial frequencies, so when we step back or squint our eyes the low frequencies dominate (typically revealing a large portrait), but when we move in close the high frequencies take over instead (usually depicting a detailed scene). (See for example *Nieuw Amsterdam, The Three Ages, Changes in Great Masterpieces, Rembrandt*, or *Transformation of Antiques Magazine Cover*).

An interactive installation named “Gala Contemplating You” was the centerpiece of the “Marvels of Illusion” exhibit at the Dali Museum. “Gala Contemplating You” replaced Lincoln's image in the *Gala Contemplating* … painting with the blocked portraits of museum visitors (See http://www.galacontemplatingyou.com/gallery/1).

## Depth perception and stereoscopic vision in Dali's art

On a flat canvas, there is no actual foreground or background: a flat picture involving perspective is a type of illusion. Since the visual system only has indirect access to depth information about its surroundings (our retinas are essentially bi-dimensional), we experience the third dimension *always* as a mental construct, both when we look at art in the museum and out in the world. Depth perception is the consequence of a set of rules, originated in neural calculations, which artists use to create compelling three-dimensional illusions in their work. These rules comprise vanishing points, size, occlusion, shading and gradation, chiaroscuro, sfumato, and the level of transparency of the atmosphere. The same rules, also called monocular cues of depth perception, drive our real world perception–which is the reason that they also apply to artworks such as Dali's scenic paintings.

In the real world, our visual system moreover relies on binocular, or stereoscopic cues to depth perception.

### Crucifixion (christ of gala)—lithograph, 1981

Stereopsis is the neural mechanism by which the visual system combines the horizontally displaced images from the left and right eye to produce a 3D percept. Dali's interest in perception led him to experiment with stereoscopic vision, creating a number of paintings as stereo-pairs. That is, he achieved three-dimensionality by creating two versions of the same scene (one for each eye of the observer, thus mimicking the horizontal disparity of binocular images in natural vision). Each painting was meticulously rendered from slightly different viewing points, equivalent to the differences that would result from viewing the same image with the right vs. the left eye, had the observer witnessed this scene in real life. Dali's adjustments to position, tone, lighting and symmetry took into account the distance between the viewer and the image.

**Figure 19:**
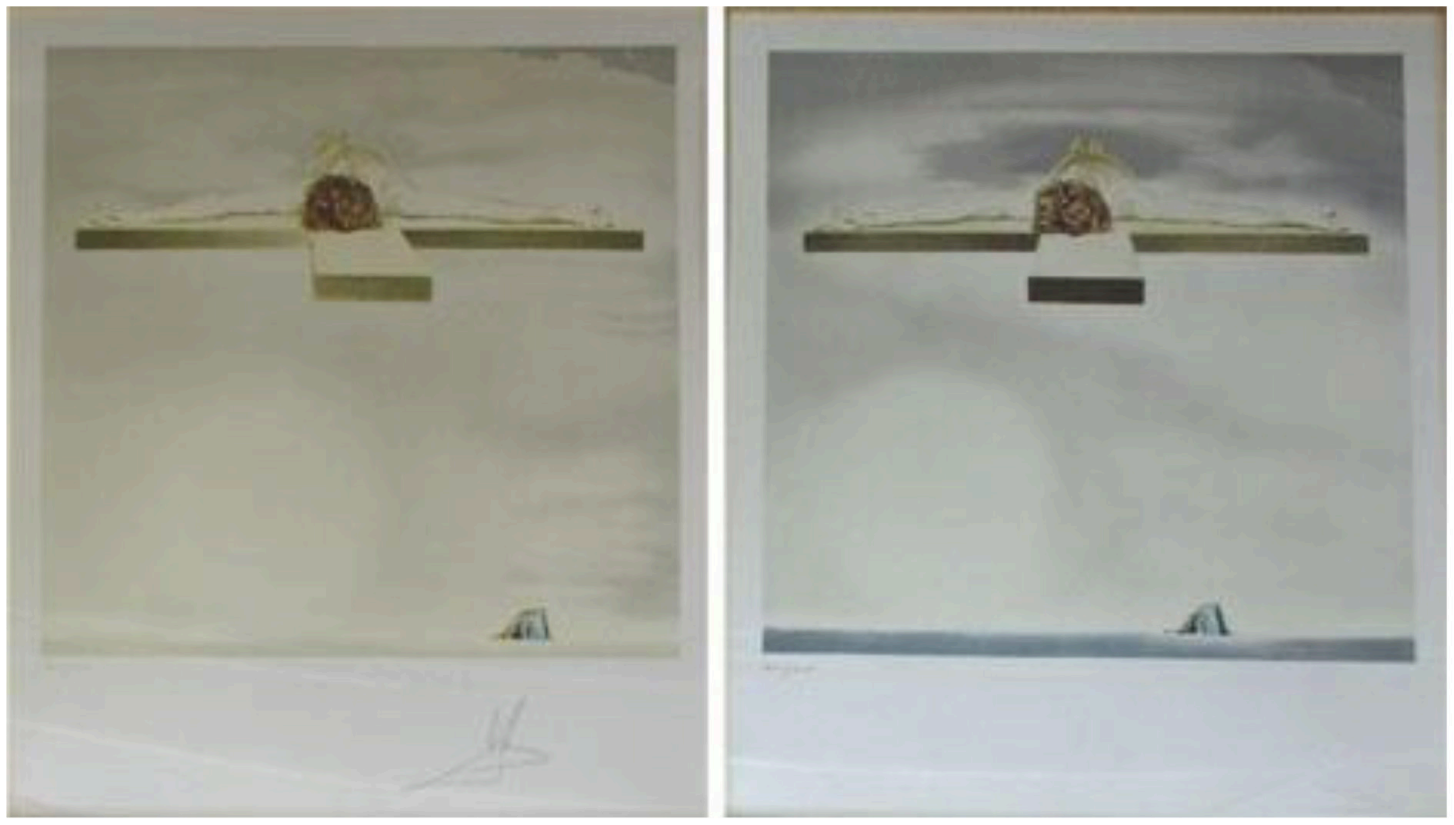
**Crucifixion (Christ of Gala), by Salvador Dali, Lithograph, 1981**. ^©^Salvador Dali. Fundación Gala-Salvador Dali (Artist Rights Society), 2015. Collection of the Salvador Dali Museum, Inc., St. Petersburg, FL, 2015. Reproduced with kind permission from the Salvador Dali Museum, Inc.

*Crucifixion* combines very effectively binocular cues (stereopsis) and monocular cues to depth perception, the latter most powerfully in the form of vanishing points (i.e., the cross appears to recede in the distance, even when we close one eye). When we observe both images side-by-side, with the left eye focused on the left picture, and the right eye on the right picture, our visual system combines both images into a single three-dimensional one (Figure 19).

### Le crane (skull)—lithograph, 1972

**Figure 20:**
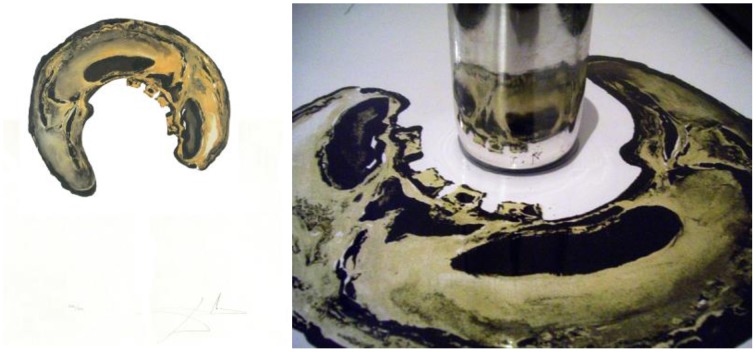
**Le Crane (Skull)**. From Anamorphoses, by Salvador Dali, Lithograph, 1972. ^©^Salvador Dali. Fundación Gala-Salvador Dali (Artist Rights Society), 2015. Collection of the Salvador Dali Museum, Inc., St. Petersburg, FL, 2015. Reproduced with kind permission from the Salvador Dali Museum, Inc.

Dali's 1972 image *Le Crane* (*Skull*), from his 1972 lithograph suite *Anamorphoses*, combines an optical illusion (the reflection of the image provided by the cylindrical mirror) with a visual illusion involving anamorphic perspective. Anamorphic images are distorted so that they are unevenly enlarged along perpendicular axes. These images are not immediately recognizable from all sides, but appear normal when viewed from a particular point, shallow angle, or with a particular lens or mirror. In *Le Crane*, what appears at first glance to be an abstract swirl is recognized in the mirror as a skull. Skulls are often used by artists as a reminder of human temporality. By hiding the skull within an abstract pattern, Dali appears to hide a secret about human nature that the viewer can unlock only by using the necessary device, the cylindrical mirror (Figure 20).

### Dix recettes d'immortalite (ten recipes of immortality)—engraving, 1973

**Figure 21:**
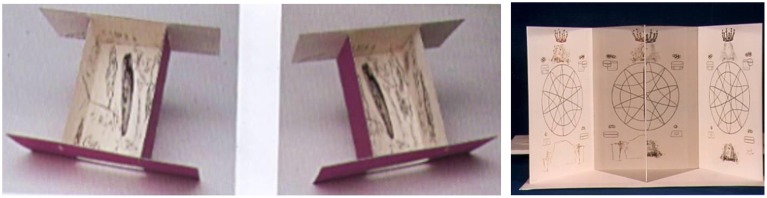
**Two examples from Dix Recettes d'Immortalite (10 Recipes of Immortality), by Salvador Dali, Engraving, 1973**. **Left and center:** Anamorphosis. **Right:** Immortal Monarchy. ^©^Salvador Dali. Fundación Gala-Salvador Dali (Artist Rights Society), 2015. Collection of the Salvador Dali Museum, Inc., St. Petersburg, FL, 2015. Reproduced with kind permission from the Salvador Dali Museum, Inc.

Whereas in *Crucifixion* the viewer must uncross his or her eyes to achieve stereovision, in *Immortal Monarchy* from the “Ten Recipes of Immortality” suite, the viewer places his or her nose at the apex of v-angled mirrors, to force each eye to see only a specific image. The visual cortex then combines the two images to perceive a three-dimensional sphere (Figure 21, right).

In the *Anamorphosis* box construction, also from the “Ten Recipes of Immortality” suite, the viewer's perception changes radically just by changing his or her visual point of view, revealing additional images including both an anamorphic skull by Dali, and a second anamorphic skull by Hans Holbein the Younger, from the 1533 painting *Les Ambassadors* (Figure 21, left and center). Through such illusions, artists from the Renaissance on have suggested a form of imagery that can only be understood by those who know its secrets.

## Conclusions

We have described how Dali made constant use of illusions in his artworks to blur the distinction between fact and fantasy, a hallmark of the surrealist movement. Illusions are—or should be—a fundamental part of the neuroscientist's toolbox to explore how the brain creates an internal representation of the external world. In addition, illusions add an intellectual dimension to the aesthetic and emotional engagement that typically characterizes the experience of art. Dali's use of illusion forces the viewer to interact with his artworks in a questioning, analytical way, so as to puzzle out what is perception vs. reality. He transforms the observer into an active practitioner of Dali's signature paranoic-critical method, by which any object can be seen as another.

### Conflict of interest statement

The authors declare that the research was conducted in the absence of any commercial or financial relationships that could be construed as a potential conflict of interest.
